# Muscle strength, muscle morphology, and oxidative capacity in normal weight versus overweight and obese youth: a systematic review with meta-analysis

**DOI:** 10.1038/s41598-025-24024-5

**Published:** 2025-10-15

**Authors:** Mauricio Inostroza-Mondaca, Omar Valdés, Rodrigo Ramirez-Campillo, Urs Granacher

**Affiliations:** 1 Departamento de Kinesiología, Laboratorio de Actividad Física, Salud y Rendimiento Humano, Universidad Metropolitana de Ciencias de la Educación, Ñuñoa, Chile; 2 Exercise and Rehabilitation Sciences Institute, School of Physical Therapy, Faculty of Rehabilitation Sciences, Universidad Andres Bello, Santiago, Chile; 3 Sport Sciences and Human Performance Laboratories, Instituto de Alta Investigación, Universidad de Tarapacá, Arica, Chile; 4 Department of Physical Activity Sciences, Universidad de Los Lagos, Osorno, Chile; 5 AME2P, Université Clermont Auvergne, 63000 Clermont-Ferrand, France; 6 Escuela de Kinesiología, Facultad de Ciencias de La Salud, Universidad de Las Américas, 2520000 Viña Del Mar, Chile; 7 Department of Sport and Sport Science, Exercise and Human Movement Science, University of Freiburg, Freiburg, Germany

**Keywords:** Pediatric obesity, Muscle function, Muscle metabolism, Muscle cross-sectional area, VO_2_ kinetics, Endocrinology, Obesity

## Abstract

The World Health Organization (WHO) defined youth overweight and obesity as pandemic due to a large global overweight and obesity prevalence of 14.8% and 8.1% in 2023. However, the impact of overweight and obesity on youth muscle strength, morphology, and oxidative capacity is unclear despite increased research efforts. To systematically review the literature and meta-analyze findings on muscle strength, morphology, and oxidative capacity in youth with overweight and/or obesity (OW) versus those with normal-weight (NW). Following PRISMA guidelines, electronic databases (i.e., PubMed, Web of Science, Scopus, CINAHL, MEDLINE) were searched for cross-sectional studies that compared OW with NW regarding the outcomes muscle strength (e.g., absolute, relative [e.g., to fat-free mass, body mass]), morphology (e.g., muscle cross-sectional area [CSA]), and oxidative capacity (e.g., phase II of volume of oxygen [VO_2_] kinetics). The risk of bias (RoB) was assessed using the Joanna Briggs Institute 8-item checklist. A random-effects three-level meta-analysis was used, and within- as well as between-group effect sizes (Hedges’ g [g]) were computed. Study heterogeneity was assessed using I^2^ statistics. The Grading Recommendation Assessment Development and Evaluation (GRADE) approach was used to evaluate the certainty of evidence. Overall, 15 studies were meta-analyzed, including 1,475 participants aged 8.4 to 16.6 years. The mean RoB amounted to 6.1 on a 0 to 8 scale. Muscle absolute strength (n = 346; g = 0.68; *p* = 0.011; I^2^ = 59.3% [*p* < 0.001]) and CSA (n = 1,024; g = 1.12; *p* < 0.001; I^2^ = 73% [*p* < 0.001]) was significantly greater in OW versus NW. However, relative muscle strength tended to be larger in NW versus OW (n = 320; g = -0.455; *p* = 0.165; I^2^ = 92.7% [*p* < 0.001]). Moreover, muscle oxidative capacity was significantly greater in NW versus OW (n = 105; g = -0.58; *p* = 0.019; I^2^ = 37.5% [*p* = 0.18]). GRADE revealed very low (absolute and relative strength and muscle oxidative capacity) to moderate (muscle CSA) certainty of evidence. Absolute muscle strength and CSA seem larger in OW versus NW, potentially related to the chronic overload during every day and sports-related tasks imposed by overweight and/or obesity. However, relative muscle strength tended to be lower in OW versus NW, suggesting impaired muscle quality in OW. Further, muscle oxidative capacity seems lower in OW versus NW, which might be indicative of muscle mitochondrial dysfunction and impaired oxygen supply, with a potential negative impact on exercise tolerance. However, the cross-sectional nature of included studies, results heterogeneity, and reduced control of confounders among the included studies (e.g., biological maturation) preclude a robust conclusion, due to a low certainty of evidence.

**Supplementary Information:**

The online version contains supplementary material available at 10.1038/s41598-025-24024-5.

## Introduction

In 2023, the global prevalence of youth with overweight and obesity reached 14.8% and 8.1%, respectively^1^. Between 1990 and 2022, obesity rates increased from 1.9 to 8.1%, which equals an increase of 63.9 million youth in absolute numbers^2^. Obesity has been defined as an abnormal or excessive body fat accumulation that increases the likelihood of sustaining adverse health events and comorbidities^3^ such as high blood pressure or cardiovascular diseases (e.g., diabetes)^4^. Accordingly, the World Health Organization (WHO) refers to youth obesity as a pandemic. Due to growth and maturation, the absolute body mass index (BMI) thresholds used in the western adult population (overweight: 25.0–29.9 kg/m^2^; obesity: ≥ 30.0 kg/m^2^) are inappropriate for youth populations. Instead, overweight and obesity have been classified in youth cohorts according to the BMI distribution across the general youth population, with overweight being defined as + 2 standard deviations (SD) from the mean and obesity as + 3 SD from the mean^5^.

There is preliminary evidence indicating that fat infiltrations may alter muscle strength^6,7^, muscle morphology^7,8^, and muscle oxidative capacity^9,10^ in overweight and obese youth. For example, youth with obesity showed greater peak torque of the knee extensors (∆60.0%) and vastus medialis muscle thickness (29.7%) compared with normal-weight peers^7^. Additionally, a lower capacity of skeletal muscles to metabolize oxygen was found in youth with obesity versus youth with normal-weight^9,10^. More specifically, Lambrick et al.^9^ and Salvadego et al.^10^ reported a slower volume of oxygen (VO_2_) kinetics phase II mean response time (∆28.5%) and time constant (∆21.5%), respectively. Taken together, the observed alterations in muscle strength, muscle morphology, and muscle oxidative capacity may lower physical activity levels^11^, enhance sedentariness, and increase the risk of sustaining musculoskeletal pathologies^12^ or exercise intolerance^13^, ultimately resulting in an impaired quality of life.

However, there is inconsistency in the literature with regard to how youth overweight and obesity affect muscular characteristics such as muscle strength, morphology, and oxidative capacity. While some authors reported greater absolute torque of the quadriceps muscle (∆4.3%)^14^, absolute maximal voluntary contraction of the dorsal interosseus muscle (∆2.6%)^15^, and absolute torque of the plantar flexors (∆5.1%)^16^ in normal-weight versus youth with obesity, other researchers reported the exact opposite^7^. Similarly, greater quadriceps muscle cross-sectional area (CSA) (∆5.6%) and dorsal interosseous muscle thickness (∆69.4%) were found in normal weight versus obese youth^14,15^. Again, findings are not uniform, and other researchers have reported the opposite results^7^. For skeletal muscle oxidative capacity, some studies reported a ∆22.4% slower phase II time constant of the VO_2_ kinetics in normal weight versus youth with obesity^17^, while others showed the opposite direction^9,10^. Over the past years, an increased number of studies have been published, yet there is no consensus regarding the impact of youth overweight and obesity on muscle strength, morphology, and oxidative capacity.

A meta-analytical summary of the available literature may provide new insights into the adverse effects of excessive body fat on muscle strength, muscle morphology, and muscle oxidative capacity in youth. Indeed, a systematic review with meta-analysis constitutes the highest evidence level^18^, and the inclusion of meta-analytical techniques such as moderator analyses (e.g., lower vs. upper limb muscles), sensibility analysis, and meta-regression may provide further insights. Therefore, the objective of this study was to systematically review the literature and meta-analyze findings on muscle strength, morphology, and oxidative capacity in normal weight versus overweight and /or obese youth.

## Methods

### Protocol and registration

This systematic review followed the Preferred Reporting Items for Systematic Reviews and Meta-Analyses (PRISMA 2020) Statement Checklist^19^ (Supplementary data 1) and was registered in PROSPERO (International Prospective Register of Systematic Reviews; identifier CRD42024529116) before the systematic literature search.

### Eligibility criteria

To be eligible for inclusion, studies had to compare markers of muscle strength, muscle morphology, and muscle oxidative capacity in youth with normal weight (NW) versus youth with overweight and/or obesity (OW). Youth with overweight and obesity were identified using BMI percentiles (> 85th percentile for overweight and > 95th for obesity, respectively)^20^. Similarly, BMI values of + 2 SD and + 3 SD from the mean for sex and age were also used to classify individuals as overweight and obese, respectively^5^. In addition to BMI and SD categorization, body fat > 30% was also used^21^. Studies were excluded if there were indications in the articles that youth patient groups were examined (e.g., type 1 diabetes, primary dyslipidemias, secondary hypertension, cystic fibrosis, genetic diseases [e.g., Prader-Willis syndrome], cerebral palsy, neuromuscular diseases [e.g., Duchenne dystrophy], congenital heart disease, and intellectual disabilities).

### Information sources

A systematic literature search was conducted with no language restrictions and hardly any filters, except studies with humans in the electronic databases PubMed, Web of Science (WoS), Scopus, MEDLINE, and CINAHL from inception up to April 2024 and updated in March 2025. Additionally, the reference list of the included studies was searched for studies that met the inclusion criteria. The complete search strategies for all databases are presented in Supplementary Data 2.

### Selection process

Two independent reviewers (MI and OV) performed the selection process by screening titles, abstracts, and full texts to determine whether the studies were eligible for inclusion. In case of disagreement, a third reviewer (RRC) was consulted.

### Data collection

For each study, two independent reviewers (MI and OV) performed the data collection. The following study characteristics were extracted: i) authors, ii) year of publication, iii) study title, iv) comparator, v) applied method to obtain the main outcomes, vi) main outcome, vii) pubertal development or biological maturation assessment, viii) characteristics of the study participants (i.e., sample size, sex, age, anthropometrics, body composition). If studies did not report the required data, the corresponding authors were contacted twice. In case we did not receive the requested information, the study was excluded. Additionally, we used the software ImageJ (National Institutes of Health, Bethesda, MD, USA) to obtain data from studies that provided data only as figures.

### Study outcomes

The following study outcomes were extracted from each included study and meta-analyzed: i) lower, upper limb muscle strength: absolute torque (Nm), absolute strength (N), relative torque (body mass [Nm/kg], fat-free mass [Nm/kg], or muscle CSA [Nm/mm^2^]) and relative strength (body mass [N/kg], fat-free mass [N/kg], or muscle CSA [N/mm^2^]). ii) lower, upper limbs muscle morphology: pennation angle (°), muscle thickness (mm), fascicle length (mm), muscle CSA (mm^2^), muscle echo-intensity (arbitrary units), and muscle subcutaneous fat thickness layer (mm), iii) muscle oxidative capacity: phase II of the VO_2_ kinetics (time constant [s], time delay [s], or mean response time [s]).

### Risk of bias assessment

Two independent reviewers (MI and OV) assessed the risk of bias (RoB), with disagreements resolved by a third reviewer (RRC). The Joanna Briggs Institute (JBI) critical appraisal 8-item checklist was used to determine the RoB, considering the cross-sectional nature of the included studies^22^. Point scores of 0–3, 4–6, and 7–8 were considered high, moderate, and low RoB, respectively.

### Summary measures and synthesis methods

Published studies in the field usually include rather small sample sizes (e.g., 10–20 participants per group)^23^. An a priori calculation (R studio 4.4.0; Posit; USA) for optimal statistical power^24^ indicated an estimated number of N = 80 needed to reach sufficient statistical power. For data to be meta-analyzed, at least ≥ 4 studies had to report the same outcome measure. Further, Hedges’*g* effect sizes were calculated to correct for small sample sizes^25^. Effect sizes were calculated for each outcome for NW and OW and pooled in a random effects model^26^. Hedges’g was classified as small (g ≤ 0.50), moderate (> 0.50–0.79), and large (g ≥ 0.80)^27^. Values > 3.0 were considered outliers, and sensitivity analyses were performed to assess the impact of outliers on the summary estimates (e.g., effect size, *p*-value, I^2^)^28^. The I^2^ statistic was used to explore study heterogeneity^26^, with cut-off values at < 25%, 25–75%, and > 75% classified as low, moderate, and high heterogeneity, respectively^29^. If a study reported a given outcome using two or more measurement methods (e.g., absolute torque at different velocities), a three-level meta-analysis was conducted^30^ to explore the variance on the three levels^30,31^. Level one explores the variance of the extracted effect sizes (i.e., variance due to inter-individual differences within a study), level two explores the variance between effect sizes extracted from the same study (i.e., variance due to inter-outcome differences within a study), and level three explores the variance between studies^31^. Study heterogeneity was assessed at levels two and three using I^2^ statistics^31^. Meta-analyses were performed using the Comprehensive Meta-Analysis software (version 4.0, USA) and are visually displayed in a forest plot. Individual study data were summarized in tables. Sub-analyses were computed for i) absolute and relative muscle strength metrics and ii) lower and upper limb muscles. Relative muscle strength was reported relative to fat-free mass (kg), body mass (kg), and muscle CSA (mm^2^). Additionally, sensitivity analyses were performed for i) muscle strength, ii) muscle morphology, and iii) muscle oxidative capacity, removing each study from the analyses and determining if the computed results changed or remained unchanged.

### Certainty of evidence

The certainty of evidence for the meta-analytical outcomes was evaluated using the Grading Recommendation Assessment Development and Evaluation (GRADE) approach^32^, which considers factors for downgrading (e.g., study design, risk of bias, heterogeneity, indirectness, imprecision, and publication bias) or upgrading (e.g., effect size, dose–response gradient, and residual confounding adjustments such as physical activity levels or maturational stage) the GRADE score^32^.

## Results

### Study selection and study characteristics

**Fig. 1:**
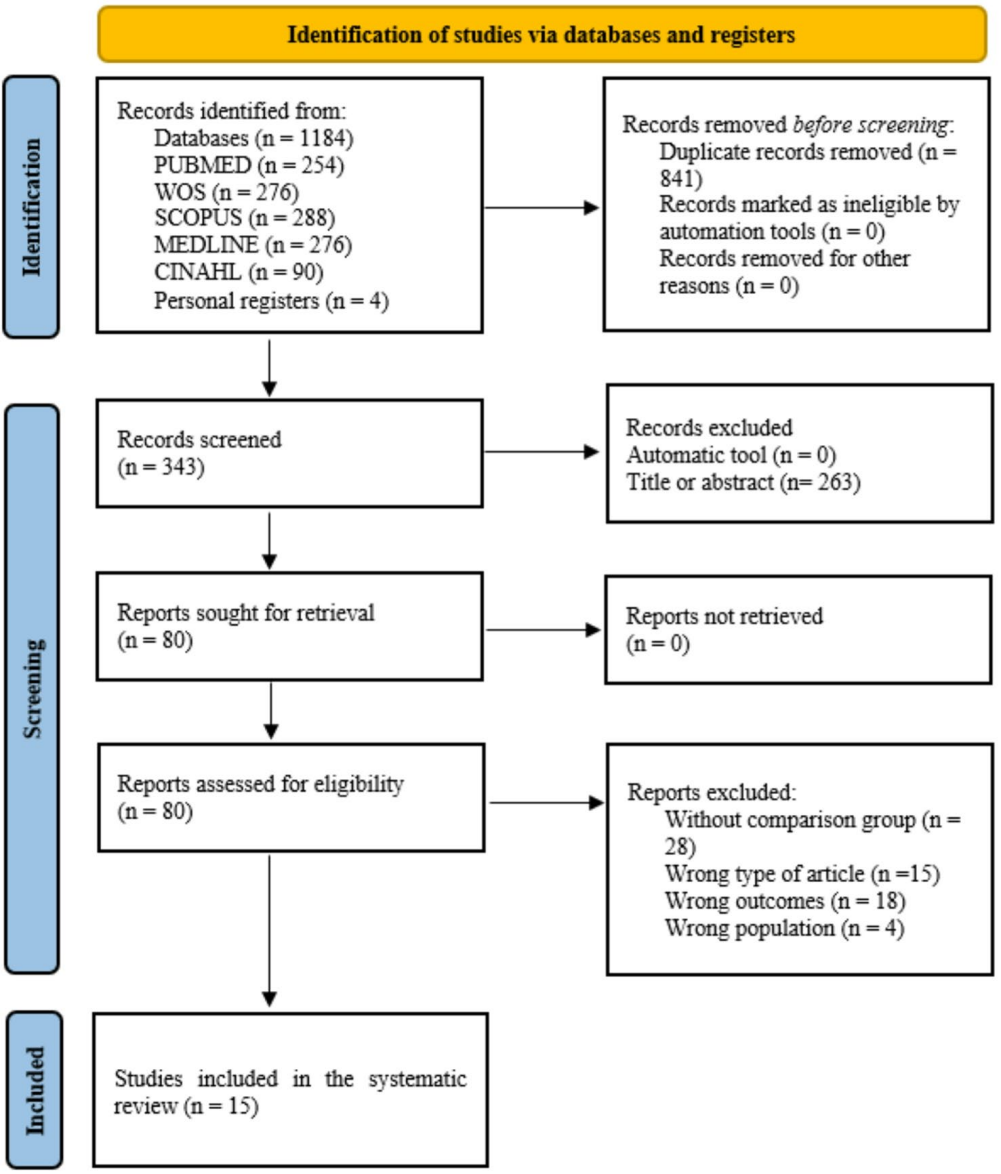
PRISMA flow diagram.

Overall, 15 studies were eligible to be included in this systematic review with meta-analysis, involving 1,475 participants (713 males and 762 females)^7,9,10,14–17,33–40^ (Fig. 1; Table 1).

**Table 1:** Summary of included articles.

Author and year	Groups sample size, biological sex, and age	Groups anthropometric characteristics (values as mean and SD)	Main outcomes comparison	RoB assessment
Ward et al. 1997^34^	**NW vs. OW** Sample size: 96 **vs.** 54 Biological sex: F = 96; M = 0 **vs.** F = 54; M = 0 Age: 10.8 ± 0.6 **vs.** 10.7 ± 0.7	**NW vs. OW** Weight: 40.9 ± 6.7 **vs.** 56.5 ± 10.9 Height: 149.0 ± 0.08 **vs.** 150.0 ± 0.06 BMI: 18.5 ± 1.9 **vs.** 25.1 ± 4.0 FFM: 33.2 ± 5.0 **vs.** 38.6 ± 4.7 Fat mass percent: 19.0 ± 4.0 **vs.** 31.0 ± 8.0	**Muscle strength** **NW vs. OW** Absolute MVC: **NW = OW** on SE and EF Relative (to BM) MVC: **NW > OW** on SE and EF Relative (to FFM) MVC: **NW = OW** on SE. **NW > OW** on EF	5 = moderate RoB
Maffiuletti et al. 2008^36^	**NW vs. OW** Sample size: 17 **vs.** 11 Biological sex: F = 5; M = 12 **vs.** F = 6; M = 5 Age: 14.9 ± 1.1 **vs.** 15.6 ± 1.2	**NW vs. OW** Weight: 56.2 ± 2.9 **vs.** 103.5 ± 2.1 Height: 170.0 ± 9.0 **vs.** 171.0 ± 6.0 BMI: 19.0 ± 1.0 **vs.** 34.0 ± 3.0 FFM: 52.9 ± 8.5 **vs.** 62.2 ± 7.4	**Muscle strength** **NW vs. OW** Absolute torque: **OW > NW** on KE Relative (to FFM) torque: **OW = NW** on KE Absolute torque at 40°: **OW > NW** on KE Relative (to FFM) torque at 40°: **OW = NW** on KE Absolute torque at 80°: **OW = NW** on KE Relative (to FFM) torque at 80°: **OW = NW** on KE	8 = low RoB
Abdelmoula et al. 2012^38^	**NW vs. OW** Sample size: 12 **vs.** 12 Biological sex: F = 0; M = 12 **vs.** F = 0; M = 12 Age: 14.4 ± 1.7 **vs.** 14.2 ± 1.4	**NW vs. OW** Weight: 53.3 ± 10.4 **vs.** 94.4 ± 16.0 Height: 165.0 ± 0.12 **vs.** 166.0 ± 0.08 BMI: 19.4 ± 1.7 **vs.** 34.1 ± 5.4 FFM: 43.9 ± 8.5 **vs.** 53.3 ± 8.5 Fat mass percent: 14.9 ± 3.7 **vs.** 40.6 ± 6.8	**Muscle strength** **NW vs. OW** Absolute torque: **OW > NW** on KE Relative (to BM) torque: **NW > OW** on KE	8 = low RoB
Blimkie et al. 1989^33^	**NW vs. OW** Sample size: 11 **vs.** 13 Biological sex: F = 0; M = 11 **vs.** F = 0; M = 13 Age: 11.1 ± 1.6 **vs.** 11.2 ± 1.2	**NW vs. OW** Weight: 38.0 ± 11.5 **vs.** 56.4 ± 13.7 Height: 147.1 ± 14.2 **vs.** 150.6 ± 7.9 BMI: 17.2 ± 1.8 **vs.** 24.6 ± 3.8 FFM: 31.7 ± 9.5 **vs.** 36.1 ± 8.4 Fat mass percent: 16.1 ± 1.7 **vs.** 35.5 ± 3.1	**Muscle strength** **NW vs. OW** Absolute isometric torque: **NW = OW** on EF and KE at 80°, 90°, 120° and 150° Absolute isokinetic torque: **NW = OW** on EF and KE at 0.52, 1.05, 2.09, and 3.14 rad Relative (to CSA) isometric torque: **NW = OW** on EF at 80°, 90°, 120°, and 150°. **NW = OW** on EF and KE at 140° and 160° Relative (to BM) isometric torque: **NW > OW** on EF at 150°. **NW = OW** on EF at 80°, 90°, and 120°. **NW > OW** on KE at 90° and 120°. **NW = OW** at 140° and 160° Relative isokinetic (to CSA) torque: **NW = OW** on EF and KE at 0.52, 1.05, 2.09, and 3.14 rad Relative isokinetic (to BM) torque: **NW = OW** on EF at 0.52, 1.05, and 2.09. **NW > OW** on KE at 3.14 rad. **NW = OW** on KE at 0.51, 1.05, 2.09, and 3.14 rad **Muscle morphology** **NW vs. OW** Muscle CSA: **NW = OW** on UA and MT	6 = moderate RoB
Blimkie et al. 1990^14^	**NW vs. OW** Sample size: 10 **vs.** 11 Biological sex: F = 0; M = 10 **vs.** F = 0; M = 11 Age: 16.5 ± 1.0 **vs.** 16.5 ± 0.9	**NW vs. OW** Weight: 63.5 ± 10.7 **vs.** 94.6 ± 14.1 Height: 180.0 ± 0.06 **vs.** 175.0 ± 0.12 BMI: 19.6 ± 2.9 **vs.** 31.0 ± 2.7 Lean mass: 55.7 ± 7.3 **vs.** 57.1 ± 20.5 Fat mass percent: 14.6 ± 3.1 **vs.** 32.3 ± 1.8	**NW vs. OW** **Muscle strength** Absolute isometric torque: **NW = OW** on KE at 80°, 90°, 120° and 150° Absolute isokinetic torque: **NW = OW** on KE at 0.51, 1.05, 2.09, and 3.14 rad Relative isometric (to BM) torque: **NW = OW** on KE at 80°, 90°, and 120°. **NW > OW** on KE at 150° Relative isokinetic (to BM) torque: **NW = OW** on KE at 0.51, 1.05, and 2.09 rad. **NW > OW** on KE at 3.14 rad **Muscle morphology** **NW vs. OW** CSA: **OW > NW** on total MT. **NW = OW** on KE	6 = moderate RoB
García-Vicencio et al. 2015^7^	**NW vs. OW** Sample size: 12 **vs.** 12 Biological sex: F = 12; M = 0 **vs. F** = 12; M = 0 Age: 13.6 ± 0.8 **vs.** 13.9 ± 0.9	**NW vs. OW** Weight: 46.9 ± 4.2 **vs.** 83.9 ± 11.6 Height: 160.0 ± 0.4 **vs.** 161.0 ± 0.9 BMI: 18.3 ± 1.3 **vs.** 32.1 ± 4.2 FFM: 35.5 ± 2.6 **vs.** 49.2 ± 4.6 Fat mass percent: 20.4 ± 2.9 **vs.** 38.2 ± 4.8 Fat mass: 9.6 ± 2.0 **vs.** 32.8 ± 4.1	**Muscle strength** **OW vs. NW** Absolute torque: **OW > NW** on KE and PF Relative (to lean mass) torque: **OW > NE** on PF **Muscle morphology** **NW vs. OW** Pennation angle: **OW > NW** on RF, VL, VM, GM, and GL Muscle thickness: **OW > NW** on RF, VL, VM, GM, and GL Muscle CSA: **OW > NW** on RF, VL, VM, and GM	8 = low RoB
Herda et al. 2018^40^	**NW vs. OW** Sample size: 14 **vs.** 15 Biological sex: F = 8; M = 6 **vs.** F = 5; M = 10 Age: 8.7 ± 0.6 **vs.** 8.8 ± 0.4	**NW vs. OW** Weight: 30.0 ± 3.1 **vs.** 40.2 ± 3.8 Height: 137.3 ± 5.4 **vs.** 138.7 ± 4.9 BMI: 15.8 ± 0.8 **vs.** 20.8 ± 1.2 BMI percentile: 40.8 ± 13.1 **vs.** 91.1 ± 4.2 Fat mass percent: 29.1 ± **vs.** 18.0 ± 3.0	**Muscle strength** **NW vs. OW** Absolute torque: **OW = NW** on KE **Muscle morphology** **NW vs. OW** Muscle CSA: **OW > NW** on VL Muscle echo-intensity: **OW > NW** on VL Subcutaneous fat thickness: **OW > NW** on VL	5 = moderate RoB
Miller et al. 2018^15^	**NW vs. OW** Sample size: 17 **vs.** 11 Biological sex: F = 5; M = 12 **vs.** F = 6; M = 5 Age: 8.8 ± 0.7 **vs.** 8.9 ± 0.9	**NW vs. OW** Weight: 30.9 ± 3.6 **vs.** 41.3 ± 7.3 Height: 139.1 ± 6.3 **vs.** 139.1 ± 7.4 BMI: 15.9 ± 0.9 **vs.** 21.2 ± 2.1 Fat mass: 17.0 ± 3.2 **vs.** 31.0 ± 4.9	**Muscle strength** **NW vs. OW** Absolute MVC: **OW = NW** on IOD Relative (to CSA) MVC: **OW = NW** on IOD **Muscle morphology** **NW vs. OW** Muscle CSA: **OW = NW** on IOD Subcutaneous fat thickness: **OW > NW** on IOD Echo intensity: **OW > NW** on IOD	5 = moderate RoB
Herda et al. 2019^16^	**NW vs. OW** Sample size: 14 **vs.** 12 Biological sex: F = 8; M = 6 **vs.** F = 6; M = 6 Age: 8.6 ± 1.1 **vs.** 8.8 ± 0.9	**NW vs. OW** Weight: 30 ± 5.4 **vs.** 42.7 ± 7.8 Height: 137.3 ± 9.4 **vs.** 143.4 ± 10.1 BMI: 15.8 ± 1.4 **vs.** 21.0 ± 2.4 Fat mass percent: 18.0 ± 5.2 **vs.** 29.3 ± 5.3	**Muscle strength** **NW vs. OW** Absolute torque: **OW = NW** on KE and PF **Muscle morphology** **NW vs. OW** Muscle CSA: **OW > NW** on VL and GM Muscle echo-intensity: **OW > NW** on VL and GM Subcutaneous fat thickness: **OW > NW** on VL and GM	6 = moderate RoB
Wetzsteon et al. 2008^35^	**NW vs. OW** Sample size: 302 **vs.** 143 Biological sex: F = 165; M = 137 **vs.** F = 54; M = 89 Age: 10.2 ± 0.6 **vs.** 10.1 ± 0.6	**NW vs. OW** Weight: 31.8 ± 4.7 **vs.** 47.4 ± 8.1 Height: 139.3 ± 6.9 **vs.** 144.1 ± 7.0 BMI: 16.3 ± 1.3 **vs.** 22.7 ± 2.6 Lean mass: 23.2 ± 3.2 **vs.** 28.6 ± 3.7 Fat mass percent: 21.6 ± 5.2 **vs.** 35.3 ± 3.5 Fat mass: 6.9 ± 2.6 **vs.** 16.2 ± 5.1	**Muscle morphology** **NW vs. OW** Muscle CSA: **OW > OW** on the lower leg	7 = low RoB
Ducher et al. 2009^37^	**NW vs. OW** Sample size: 334 **vs.** 93 Biological sex: F = 170; M = 164 **vs.** F = 54; M = 89 Age: 8.4 ± 0.4 **vs.** 8.4 ± 0.4	**NW vs. OW** Weight: 27.2 ± 3.5 **vs.** 37.3 ± 5.8 Height: 129.9 ± 5.9 **vs.** 133.0 ± 6.2 BMI: 16.1 ± 1.2 **vs.** 21.0 ± 2.1	**Muscle morphology** **NW vs. OW** Muscle CSA: **OW > OW** on the forearm and lower leg	4 = moderate RoB
Loftin et al. 2005^17^	**NW vs. OW** Sample size: 10 **vs.** 8 Biological sex: F = 10; M = 0 **vs.** F = 8; M = 0 Age: 14.4 ± 3.0 **vs.** 16.3 ± 2.8	**NW vs. OW** Weight: 55.3 ± 7.0 **vs.** 90.5 ± 18.0 Height: 148.0 ± 4.0 **vs.** 149.0 ± 9.0 BMI: 25.2 ± 2.5 **vs.** 41.2 ± 9.2 FFM: 40.2 ± 4.5 **vs.** 45.5 ± 6.2 Fat mass: 14.4 ± 3.3 **vs.** 39.5 ± 2.8 Fat mass percent: 27.4 ± 3.9 **vs.** 47.9 ± 4.9	**Muscle oxidative capacity** **NW vs. OW** Phase II time constant: **NW > OW**	3 = high RoB
Salvadego et al. 2010^10^	**NW vs. OW** Sample size: 13 **vs.** 14 Biological sex: F = 0; M = 13 **vs.** F = 0; M = 14 Age: 16.6 ± 1.1 **vs.** 16.5 ± 1.0	**NW vs. OW** Weight: 62.2 ± 10.1 **vs.** 103.2 ± 10.0 Height: 173.0 ± 11.1 **vs.** 173.0 ± 8.0 BMI: 22.0 ± 2.6 **vs.** 34.5 ± 3.1 FFM: 55.4 ± 7.9 **vs.** 63.6 ± 6.3 Fat mass: 10.8 ± 3.1 **vs.** 39.5 ± 5.5 Fat mass percent: 16.2 ± 3.4 **vs.** 27.4 ± 3.9	**Muscle oxidative capacity** **NW vs. OW** Phase II time constant: **OW > NW** at MI Phase II time delay: **NW = OW** at MI Phase II amplitude: **NW = OW** at MI Phase II time constant: **OW > NW** at HI Phase II time delay: **NW = OW** at HI Phase II amplitude: **NW = OW** at HI GET at VO_2_: **NW = OW** GET: **NW > OW**	8 = low RoB
Lambrick et al. 2013^9^	**NW vs. OW** Sample size: 19 **vs.** 18 Biological sex: F = 9; M = 10 **vs.** F = 9; M = 9 Age: 9.7 ± 0.5 **vs.** 9.8 ± 0.5	**NW vs. OW** Weight: 35.0 ± 3.5 **vs.** 51.5 ± 7.0 Height: 140.0 ± 6.1 **vs.** 145.7 ± 6.9 BMI: 17.6 ± 1.0 **vs.** 24.1 ± 2.0	**Muscle oxidative capacity** **NW vs. OW** Phase II time constant: **OW > NW** at MI Phase II time delay: **NW > OW** at MI Phase II mean response time: **OW > NW** at MI Phase II amplitude: **NW = OW** at MI Phase II time constant: **OW > NW** at HI Phase II time delay: **NW > OW** at HI Phase II mean response time: **OW > NW** at HI Phase II amplitude: **OW > NW** at HI GET at VO_2_: **NW = OW** GET (L/min): **NW = OW**	5 = moderate RoB
Potter et al. 2013^39^	**NW vs. OW** Sample size: 12 **vs.** 11 Biological sex: F = 7; M = 5 **vs.** F = 5; M = 6 Age: 11.9 ± 0.4 **vs.** 11.9 ± 0.8	**NW vs. OW** Weight: 39.4 ± 5.8 **vs.** 62.9 ± 9.7 Height: 147.0 ± 5.0 **vs.** 158.0 ± 5.0 BMI: 18.3 ± 1.8 **vs.** 25.8 ± 3.4	**Muscle oxidative capacity** **NW vs. OW** Phase II time constant: **OW > NW** at MI Phase II time delay: **NW = OW** at MI Phase II mean response time: **OW > NW** at MI Phase II amplitude: **NW = OW** at MI GET at VO_2_: **NW = OW** GET: **NW = OW**	6 = moderate RoB

BM, body mass; BMI, Body Mass Index; CSA, cross-sectional area; EF, elbow flexors; F, female; FFM, fat-free mass; GET, gas exchange threshold; GL, gastrocnemius lateralis; GM, gastrocnemius medialis; HI, high intensity; IOD, interosseus dorsalis; KE, knee extensors; M, male; MI, moderate intensity; MT, mid tight; MVC, maximal voluntary contraction; NW, normal weight; PF, plantar flexors; RF, rectus femoris; RoB, risk of bias; SD, standard deviation; SE, shoulder extension; TS, triceps surae; OW, overweight or obese; UA, upper arm; VL, vastus lateralis; VM, vastus medialis: VO_2_, maximal oxygen consumption.

*The studies were first categorized based on their outcomes in the following order: (1) studies with outcomes related only to muscle strength, (2) studies that share outcomes of muscle strength and morphology, (3) studies with outcomes related only to muscle morphology, and (4) studies with outcomes related only to oxidative muscle capacity. Within each category, the studies were then arranged by year of publication.

### Risk of bias

The mean RoB score was 6.1 (from a maximum possible of 8 points) for the 15 studies included in the meta-analysis (Table 1).

### Muscle strength

**Fig. 2:**
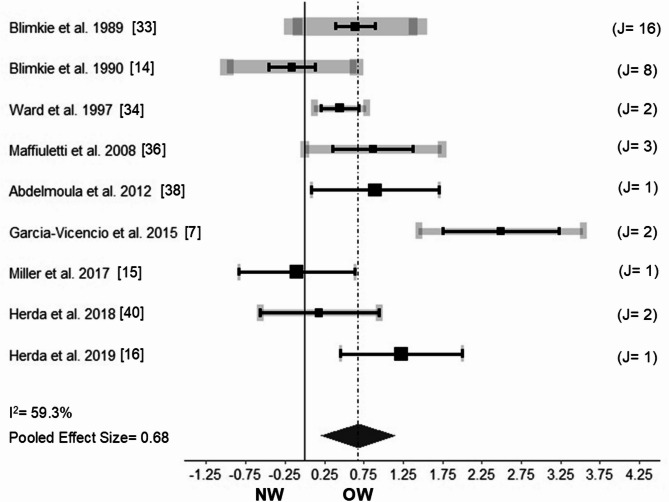
Forest plot showing the pooled Hedges’g effect size and heterogeneity (I^2^) for absolute muscle strength between youth with normal-weight (NW) and overweight and/or obesity (OW). J: denotes the number of outcomes that each study provided for the analysis. The number is in line with the horizontal gray bar thickness, and with the horizontal gray bar length (variance of the effect size for a given study). For example, J = 2 may denote that the outcome of absolute muscle strength of the gastrocnemius medialis muscle and vastus lateralis muscle provided data for the Hedges’g effect size calculated for a given study. The black squares and the horizontal black bars represent studies mean effect size and 95% confidence interval, respectively.

Absolute muscle strength was measured in studies involving males^14,33,36,38^, females^7,34^, and males and females combined^15,16,40^ for a total of 196 NW (67 males and 129 females) participants aged 8.6 to 16.5 years, and 150 OW (67 males and 83 females) participants aged 8.8 to 16.6 years. Absolute muscle strength was significantly greater in OW compared to NW (Fig. 2; Hedges´g = 0.68; CI 95% 0.164–1.207; *p*-value = 0.011; I^2^ = 59.3% [*p*-value < 0.001]). The three-level meta-analysis showed that the major source of heterogeneity occurred on level three (*p* < 0.001), with variances for levels one, two, and three at 21.7%, 4.1%, and 78.2%, respectively. The computed sub-analyses revealed that absolute muscle strength was significantly greater in OW compared to NW for the lower (g = 0.88; *p* < 0.001) and upper limb muscles (g = 0.52; *p* = 0.034). The removal of one study^7^ during sensitivity analysis resulted in a lower Hedges’g effect size (g = 0.45; between-groups *p* = 0.08).

Relative muscle strength (relative to fat-free mass, body mass, or muscle CSA) was measured in studies involving males^14,33,36,38^, females^7,34^, and males and females combined^15,40^, for a total of 182 NW (61 males and 121 females) participants aged 8.6 to 16.5 years and 138 OW (61 males and 67 females) participants aged 8.8 to 16.6 years. Although the analysis did not reveal statistically significant between-groups difference, relative muscle strength tended to be greater in NW versus OW (Hedges’g = -0.455; CI 95% -1.082 to 0.165; *p* = 0.165; I^2^ = 92.7% [*p* < 0.001]), particularly when strength was normalized to muscle CSA (g = -0.944; I^2^ = 74.1%). Moreover, the results remained consistent after sensitivity analyses, although the removal of one study^7^ led to g = -0.60 (p = 0.06), favoring NW. The three-level meta-analysis showed significant heterogeneity on levels two (*p* < 0.001) and three (*p* < 0.001), with variances for levels one, two, and three at 11.1%, 41.1%, and 47.9%, respectively. Subgroup analyses showed no statistically significant differences in lower/upper limb muscle strength in OW vs NW.

### Muscle morphology

**Fig. 3:**
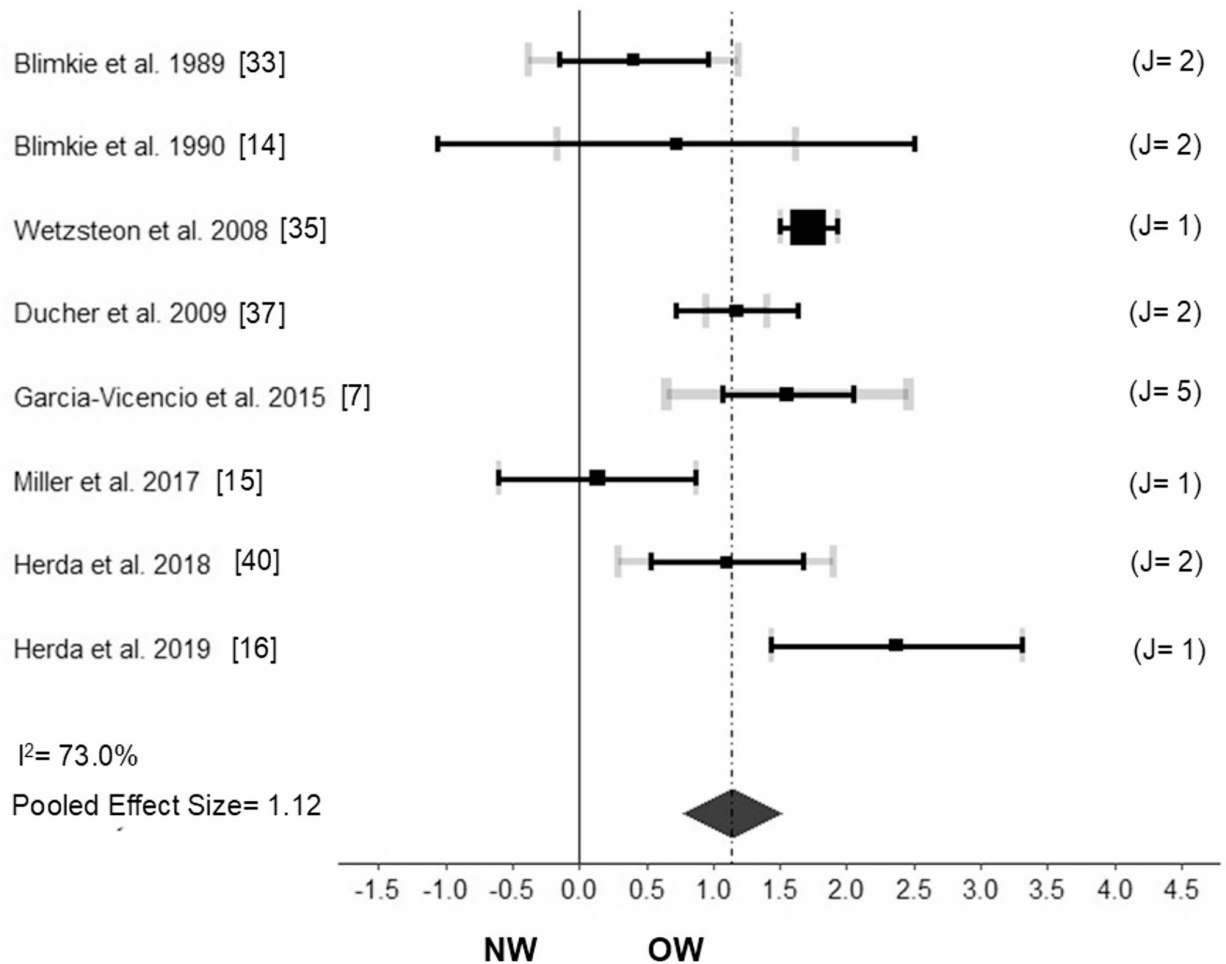
Forest plot showing the estimated pooled Hedges’g effect size and heterogeneity (I^2^) for muscle morphology (as muscle cross-sectional area) between youth with normal-weight (NW) and overweight and/or obese (OW). J: denotes the number of outcomes that each study provided for the analysis. The number is in line with the horizontal gray bar thickness, and with the horizontal gray bar length (variance of the effect size for a given study). For example, J = 2 may denote that the outcome cross-sectional area of the gastrocnemius lateralis muscle and the outcome cross-sectional area of the gastrocnemius medialis muscle provided data for the Hedges’g effect size calculated for a given study. The black squares and horizontal black bars represent the studies’ mean effect size and 95% confidence interval, respectively.

Eight studies measured muscle morphology (i.e., muscle CSA)^7,14–16,33,35,37,40^ in a total of 714 NW (352 males, 362 females; aged 8.4 to 16.5 years) and 310 OW (170 males, 140 females; aged 8.3 to 16.5 years). Two studies^14,33^ included males, one study^7^ included females, and five studies^15,16,35,37,40^ included both males and females. Muscle CSA was significantly greater in OW versus NW (Fig. 3: Hedges’g = 1.12; CI 95% 0.658–1.158; *p* < 0.001; I^2^ = 73.0% [*p* < 0.001]). The three-level meta-analysis showed no significant heterogeneity in levels two and three, with variances for levels one, two, and three at 27.3%, 32.6%, and 39.9%, respectively. The results remained robust after sensitivity analyses (i.e., no changes in Hedges’g, p-value, and I^2^).

### Muscle oxidative capacity

**Fig. 4:**
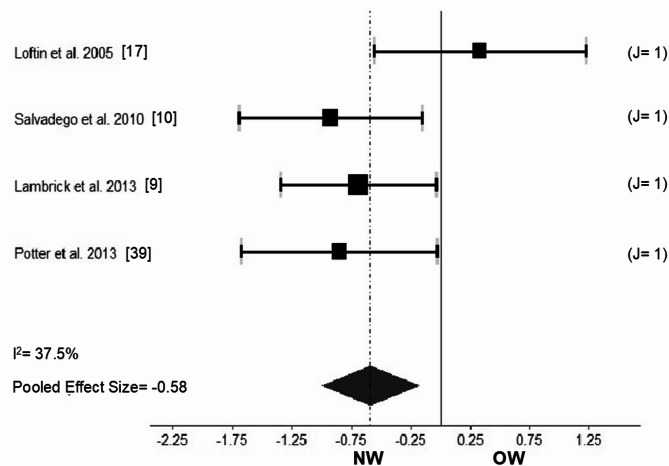
Forest plot comparing muscle oxidative capacity (as phase II time constant of the VO_2_ kinetics) between youth with normal-weight (NW) and overweight and/or obesity (OW). J: denotes the number of outcomes that each study provided for the analysis. The number is in line with the horizontal gray bar thickness, and with the horizontal gray bar length (variance of the effect size for a given study). For example, J = 1 may denote only the outcome of the phase II time constant of the VO_2_ kinetics at moderate intensity provided data for the Hedges’g effect size calculated for a given study. The black squares and horizontal black bars represent the studies’ mean effect size and 95% confidence interval, respectively.

Muscle oxidative capacity expressed as the phase II time constant of the VO_2_ kinetics was measured in studies involving males^17^, females^10^, and males and females combined^9,39^, involving 54 NW participants (28 males and 26 females) aged 9.7 to 16.6 years, and 51 OW participants (29 males and 22 females) aged 9.3 to 16.5 years. Compared with OW, NW showed a significantly faster phase II time constant of the VO_2_ kinetics (Fig. 4) (Hedges’g = -0.58; CI 95% -1.061 to -0.093; *p* = 0.019; I^2^ = 37.5% [*p* = 0.18]). The results remained consistent after sensitivity analysis. The removal of one study^17^ led to g = -0.80 and I^2^ = 0.0%.

### Certainty of evidence

The GRADE score (Table 2) revealed moderate to very low certainty of evidence.

**Table 2:** Outcome-level certainty of evidence (GRADE).

Outcome	Studies (n)	Reports (n)	Participants (n)	Hedges’g ES	GRADE
Absolute muscle strength	9	36	346	0.69	Very low ^c, d, e^
Relative muscle strength	8	40	320	-0.45	Very low ^b, c^
Muscle morphology (muscle cross-sectional area)	8	16	1024	1.12	Moderate ^d, e^
Muscle oxidative capacity (phase II time constant of VO_2_ kinetics)	4	4	105	-0.58	Very low ^a, c^

Reasons for downgrade: ^a^Risk of bias; ^b^Inconsistency of results; ^c^Imprecision; ^d^Publication bias assessed by Egger test.

Reasons for upgrade: ^e^Magnitude of effect as Hedges’g effect size (ES).

## Discussion

Findings from this meta-analysis revealed significantly greater absolute (not relative) muscle strength, greater skeletal muscle CSA, and slower VO_2_ kinetics (i.e., reduced muscle oxidative capacity) in OW versus NW.

Larger absolute maximal strength was noted in OW compared to NW, particularly in lower limb muscles. Indeed, the six meta-analyzed studies^7,14,16,36,38,40^ showed greater (g = 0.88) lower limb muscle strength in OW compared to NW, while the magnitude of this difference was smaller for upper limb muscles (g = 0.52). This might be related to the extra body mass that OW have to carry during every day and sports-related tasks, which may act as a chronic overload stimulus^6,8,36,41^. Additionally, muscle CSA was larger in OW compared to NW (g = 1.12), and muscles with larger muscle CSA can produce greater strength at the same muscle length and contraction velocities than muscles with smaller CSA^42,43^. However, it is uncertain if greater absolute maximal strength values assessed in OW assessed in laboratory-based isometric^7,14–16,33,34,36,38,40^ or isokinetic^14,33,36^ strength tests may transfer towards every day or sports-related activities such as jumping, running, or sprinting^44^. Indeed, findings from an umbrella review indicated that jumping, running, and agility actions were impaired in youth with obesity compared to normal-weight peers^44^. Therefore, future studies in OW are needed to determine the role of absolute maximal strength compared to other factors (e.g., body fat mass) on performance in every day and sport-related activities.

Contrary to absolute muscle strength, OW presented lower (although non-significant) relative muscle strength compared to NW (g = -0.455). Similar results were reported when absolute strength was normalized to fat-free mass (g = -0.347), body mass (g = -0.561), and muscle CSA (g = -0.944). The capacity to generate muscle strength relative to body mass or CSA is related to muscle quality^45^, with important clinical implications for youth^46,47^, including the determination of cut-off points to assess sarcopenic obesity^46^, a term that has previously been used in the context of geriatric populations only and that has recently been introduced to the pediatric literature as well. Additionally, adequate development of the central nervous system in youth seems to be linked to muscle quality^48^, suggesting relative strength measures (particularly at the muscle CSA level) as a relevant marker to be assessed during youth maturation and growth processes.

Individuals with obesity have attenuated muscle anabolic signaling after feeding or exercise^49^. Moreover, prepubertal youth exhibit insufficient circulating anabolic hormones to develop muscle hypertrophy^50–54^. Of note, 96.4% (n = 299) of the included OW individuals in this meta-analysis were pre-age to peak height velocity, ≤ Tanner stage III, and/or ≤ age 9 years, suggesting an immature anabolic milieu^14–16,33,35,37^. Therefore, it was somewhat surprising that this meta-analysis (Fig. 3) revealed greater (g = 1.12, large; moderate certainty of evidence) muscle CSA in OW versus NW. However, muscle CSA increase in OW might be secondary to skeletal muscle fat infiltration (i.e., *pseudo hypertrophy*)^15^. Nonetheless, the gold standard to assess skeletal muscle fat infiltration is the muscle biopsy^55^. Thus, ethical reasons often preclude studies in youth populations. Future studies, however, may assess skeletal muscle fat infiltration and muscle CSA using valid non-invasive techniques such as magnetic resonance imaging^56^, computer tomography^55^, or ultrasound^57^.

Muscle oxidative capacity, expressed as phase II of the VO_2_ kinetics, was faster in NW versus OW. The lower, and thus impaired, muscle oxidative capacity^58^ might be related to altered fiber type distribution, mitochondrial function and content, and muscle oxygen delivery^59–64^. For example, larger type II and smaller type I CSA were found in participants with obesity compared to their normal-weight peers^60^. Further, excessive body fat may lower the mitochondrial respiratory index and efficiency^61,62^, in line with a compensatory increase in mitochondrial DNA and a lower citrate synthase content^62^. Likewise, youth with obesity showed reduced muscle oxygen delivery due to musculoskeletal microvascular dysfunction^63,64^. To compensate for reduced muscle oxidative capacity, increased glycolytic activity (capacity) can be anticipated. However, compared to adults with obesity, youth with obesity exhibit less type II muscle fibers^65^, lower glycolytic enzyme activity (e.g., phosphofructokinase)^66^, and glycogen depots^67^, thus an impaired glycolytic capacity, particularly due to a not fully developed system^65–67^. Therefore, muscle oxidative capacity in OW might be critical to tolerate exercise demands^58,68^. Future intervention studies should focus on improving OW maximal oxygen consumption^69,70^, by specifically targeting peripheral performance determinants (e.g., muscle oxidative capacity)^71^.

In line with previous narrative reviews^72,73^, this systematic review with meta-analysis confirms the impact of youth obesity on measurements of muscle strength and muscle morphology. Further, this systematic review with meta-analysis provides novel information on the effects of youth obesity on lower and upper-limb muscle strength and morphology, and muscle oxidative capacity. Moreover, the direction and magnitude of the effect of youth obesity on muscle strength, morphology, and oxidative capacity were calculated with robust-advanced statistical procedures, e.g., three-level meta-analyses. This meta-analytical technique adjusts the overall effect size by considering variance at different levels reflecting methodological variations, providing an accurate estimate by modeling sources of heterogeneity^30,31^.

Nonetheless, this systematic review with meta-analysis is not without limitations that should be acknowledged. Firstly, the cross-sectional nature of the included studies precludes cause-effect confirmation. For example, the greater muscle CSA in OW individuals might be related to pseudohypertrophy. To elucidate such possibility, researchers for future studies should include more advanced mechanistic and physiological measures, such as intramuscular lipid content using non-invasive resonance imaging^56^, computer tomography^55^, or ultrasound^57^, alongside muscle protein synthesis measures through minimally invasive methods (e.g., deuterium enrichment patterns)^74^. Similarly, researchers should assess blood biomarkers (minimally invasive) of mitochondrial function^75^ (e.g., muscle oxidative capacity) in future studies with OW youth. Moreover, the phenomenon of greater muscle strength in OW needs in-depth research to better understand the underlying mechanisms. Indeed, if stronger muscles do not develop in line with stronger tendons, the risk of tendinopathies^12^ or growth plate diseases (e.g., Sever disease or Osgood Schlatter)^76,77^ may increase.

Second, the GRADE assessment identified imprecision (e.g., small sample sizes) and publication bias (e.g., significant Egger test) for absolute muscle strength, inconsistency (e.g., high heterogeneity) and imprecision for relative muscle strength, and both risk of bias and imprecision for muscle oxidative capacity. To address these methodological limitations, researchers for future studies should include larger and adequately powered study samples and well-developed and more rigorous study designs. In addition to avoid publication bias, all findings, regardless of statistical significance level should be published. Moreover, future studies are advised to control factors that might alter the effect of youth obesity on muscle strength, morphology, and oxidative capacity, such as biological maturation^78–80^, physical activity levels^81,82^, and socioeconomic status^83^.

## Conclusions

Absolute muscle strength and CSA seem larger in OW versus NW, potentially related to the chronic overload during every day and sports-related tasks imposed by overweight and/or obesity. However, relative muscle strength tended to be lower in OW versus NW, suggesting impaired muscle quality in OW. Further, muscle oxidative capacity seems lower in OW versus NW, which might be indicative of muscle mitochondrial dysfunction and impaired oxygen supply, with a potential negative impact on exercise tolerance. However, the cross-sectional nature of included studies, results heterogeneity, and reduced control of confounders among the included studies (e.g., biological maturation) preclude a robust conclusion, due to a low certainty of evidence.

## Supplementary Information

Below is the link to the electronic supplementary material.

Supplementary Material 1

Supplementary Material 2

## Data Availability

The authors will make the data supporting this study’s findings available and securely store them. If unpublished data are available, they will be considered and provided upon reasonable request.
