# Supplementary material for: Muscle strength, muscle morphology, and oxidative capacity in normal weight versus overweight and obese youth: a systematic review with meta-analysis
Source: Sci Rep. 2025 Oct 15;15:36108. doi: 10.1038/s41598-025-24024-5 (PMC12528735; doi:10.1038/s41598-025-24024-5)
Supplement: Supplementary file 2 — Supplementary Material 2 [file 41598_2025_24024_MOESM2_ESM.docx]

Supplementary Table 1. Search Strategy.

| **Database** | **Search strategy** |
| --- | --- |
| PUBMED | (((((("VO2 kinetics"[Title/Abstract] OR "oxygen uptake kinetics"[Title/Abstract] OR "Time constant"[Title/Abstract] OR "Muscle O2 utilization"[Title/Abstract] OR "Oxidative metabolism"[Title/Abstract] OR "Muscle fatigability"[Title/Abstract] OR "Time to task failure"[Title/Abstract] OR Deoxyhemoglobin[Title/Abstract] OR "Muscle deoxygenation"[Title/Abstract] OR "Muscle oxygen utilization"[Title/Abstract] OR "Exercise Recovery"[Title/Abstract] OR "Muscle fatigue"[Title/Abstract])) OR (("Muscle architecture"[Title/Abstract] OR "Muscle pennation angle"[Title/Abstract] OR "Muscle fascicle length"[Title/Abstract] OR "Muscle function"[Title/Abstract] OR "Muscle cross-sectional area"[Title/Abstract] OR "Muscle thickness"[Title/Abstract] OR "Muscle echo-intensity"[Title/Abstract] OR "Muscle size"[Title/Abstract] OR "Rate of force development"[Title/Abstract] OR "Maximal isometric voluntary contraction"[Title/Abstract] OR "Rate of torque development"[Title/Abstract]))) AND ((Pediatric[Title/Abstract] OR Children[Title/Abstract] OR "Children and adolescents"[Title/Abstract] OR Childhood[Title/Abstract] OR Youth[Title/Abstract] OR Boys[Title/Abstract] OR Girls[Title/Abstract] OR Adolescen*[Title/Abstract] OR Maturation[Title/Abstract] OR "Peak height velocity"[Title/Abstract]))) AND ((Obese[Title/Abstract] OR Obesity[Title/Abstract] OR Overweight[Title/Abstract] OR "Body mass index"[Title/Abstract] OR "Body composition"[Title/Abstract] OR "Central obesity"[Title/Abstract] OR "Body weight"[Title/Abstract]))) |
| WOS | (((((((((TI=(("VO2 kinetics" OR "oxygen uptake kinetics" OR "Time constant" OR "Muscle O2 utilization" OR "Oxidative metabolism" OR "Muscle fatigability" OR "Time to task failure" OR Deoxyhemoglobin OR "Muscle deoxygenation" OR "Muscle oxygen utilization" OR "Exercise Recovery" OR "Muscle fatigue"))) OR TI=(("Muscle architecture" OR "Muscle pennation angle" OR "Muscle fascicle length" OR "Muscle function" OR "Muscle cross-sectional area" OR "Muscle thickness" OR "Muscle echo-intensity" OR "Muscle size" OR "Rate of force development" OR "Maximal isometric voluntary contraction" OR "Rate of torque development"))) AND TI=((Pediatric OR Children OR "Children and adolescents" OR Childhood OR Youth OR Boys OR Girls OR Adolescen* OR Maturation OR "Peak height velocity"))) AND TI=((Obese OR Obesity OR Overweight OR "Body mass index" OR "Body composition" OR "Central obesity" OR "Body weight"))) OR AB=(("VO2 kinetics" OR "oxygen uptake kinetics" OR "Time constant" OR "Muscle O2 utilization" OR "Oxidative metabolism" OR "Muscle fatigability" OR "Time to task failure" OR Deoxyhemoglobin OR "Muscle deoxygenation" OR "Muscle oxygen utilization" OR "Exercise Recovery" OR "Muscle fatigue"))) OR AB=(("Muscle architecture" OR "Muscle pennation angle" OR "Muscle fascicle length" OR "Muscle function" OR "Muscle cross-sectional area" OR "Muscle thickness" OR "Muscle echo-intensity" OR "Muscle size" OR "Rate of force development" OR "Maximal isometric voluntary contraction" OR "Rate of torque development"))) AND AB=((Pediatric OR Children OR "Children and adolescents" OR Childhood OR Youth OR Boys OR Girls OR Adolescen* OR Maturation OR "Peak height velocity"))) AND AB=((Obese OR Obesity OR Overweight OR "Body mass index" OR "Body composition" OR "Central obesity" OR "Body weight")))) |
| SCOPUS | TITLE-ABS ( ( "VO2 kinetics" OR "oxygen uptake kinetics" OR "Time constant" OR "Muscle O2 utilization" OR "Oxidative metabolism" OR "Muscle fatigability" OR "Time to task failure" OR deoxyhemoglobin OR "Muscle deoxygenation" OR "Muscle oxygen utilization" OR "Exercise Recovery" OR "Muscle fatigue" ) OR ( "Muscle architecture" OR "Muscle pennation angle" OR "Muscle fascicle length" OR "Muscle function" OR "Muscle cross-sectional area" OR "Muscle thickness" OR "Muscle echo-intensity" OR "Muscle size" OR "Rate of force development" OR "Maximal isometric voluntary contraction" OR "Rate of torque development" ) AND ( pediatric OR children OR "Children and adolescents" OR childhood OR youth OR boys OR girls OR adolescen* OR maturation OR "Peak height velocity" ) AND ( obese OR obesity OR overweight OR "Body mass index" OR "Body composition" OR "Central obesity" OR "Body weight" ) ) AND ( LIMIT-TO ( EXACTKEYWORD , "Human" ) ) |
| MEDLINE and CINAHL | TI ( ("VO2 kinetics" OR "oxygen uptake kinetics" OR "Time constant" OR "Muscle O2 utilization" OR "Oxidative metabolism" OR "Muscle fatigability" OR "Time to task failure" OR Deoxyhemoglobin OR "Muscle deoxygenation" OR "Muscle oxygen utilization" OR "Exercise Recovery" OR "Muscle fatigue" OR "Muscle architecture" OR "Muscle pennation angle" OR "Muscle fascicle length" OR "Muscle function" OR "Muscle cross-sectional area" OR "Muscle thickness" OR "Muscle echo-intensity" OR "Muscle size" OR "Rate of force development" OR "Maximal isometric voluntary contraction" OR "Rate of torque development" )) AND TI ( (Pediatric OR Children OR "Children and adolescents" OR Childhood OR Youth OR Boys OR Girls OR Adolescen* OR Maturation OR "Peak height velocity") ) AND TI ( (Obese OR Obesity OR Overweight OR "Body mass index" OR "Body composition" OR "Central obesity" OR "Body weight") ) OR AB ( ("VO2 kinetics" OR "oxygen uptake kinetics" OR "Time constant" OR "Muscle O2 utilization" OR "Oxidative metabolism" OR "Muscle fatigability" OR "Time to task failure" OR Deoxyhemoglobin OR "Muscle deoxygenation" OR "Muscle oxygen utilization" OR "Exercise Recovery" OR "Muscle fatigue" OR "Muscle architecture" OR "Muscle pennation angle" OR "Muscle fascicle length" OR "Muscle function" OR "Muscle cross-sectional area" OR "Muscle thickness" OR "Muscle echo-intensity" OR "Muscle size" OR "Rate of force development" OR "Maximal isometric voluntary contraction" OR "Rate of torque development") ) AND AB ( (Pediatric OR Children OR "Children and adolescents" OR Childhood OR Youth OR Boys OR Girls OR Adolescen* OR Maturation OR "Peak height velocity") ) AND AB ( (Obese OR Obesity OR Overweight OR "Body mass index" OR "Body composition" OR "Central obesity" OR "Body weight") ) |
